# Evaluating medical convenience in ethnic minority areas of Southwest China via road network vulnerability: a case study for Dehong autonomous prefecture

**DOI:** 10.1186/s12939-017-0702-z

**Published:** 2017-11-28

**Authors:** Xiaoyan Wei, Xuejun Liu, Liang Cheng, Lele Sun, Yingying Pan, Wenwen Zong

**Affiliations:** 1 Key Laboratory of Virtual Geographic Environment (Nanjing Normal University), Ministry of Education, Nanjing, 210023 China; 2 State Key Laboratory Cultivation Base of Geographical Environment Evolution (Jiangsu Province), Nanjing, 210023 China; 3 Jiangsu Center for Collaborative Innovation in Geographical Information Resource Development and Application, Nanjing, 210023 China; 4 Yunnan Provincial Archives of Surveying and Mapping, Kunming, 650034 China; 5 Yunnan Provincial Geomatics Centre, Kunming, 650034 China; 6 Collaborative Innovation Center for the South Sea Studies, Nanjing University, Nanjing, China; 7 Jiangsu Provincial Key Laboratory of Geographic Information Science and Technology, Nanjing University, Nanjing, China; 8 Collaborative Innovation Center of Novel Software Technology and Industrialization, Nanjing University, Nanjing, China; 9 Department of Geographic Information Science, Nanjing University, Nanjing, China; 10 School of Tourism and Geographical Sciences, Yunnan Normal University, Kunming, 650500 China

**Keywords:** Ethnic minority areas, Medical convenience, Road network, Vulnerability, Mountainous areas

## Abstract

**Background:**

Southwest China is home to more than 30 ethnic minority groups. Since most of these populations reside in mountainous areas, convenient access to medical services is an important metric of how well their livelihoods are being protected.

**Methods:**

This paper proposes a medical convenience index (MCI) and computation model for mountain residents, taking into account various conditions including topography, geology, and climate. Data on road networks were used for comprehensive evaluation from three perspectives: vulnerability, complexity, and accessibility. The model is innovative for considering road network vulnerability in mountainous areas, and proposing a method of evaluating road network vulnerability by measuring the impacts of debris flows based on only links. The model was used to compute and rank the respective MCIs for settlements of each ethnic population in the Dehong Dai and Jingpo Autonomous Prefecture of Yunnan Province, in 2009 and 2015. Data on the settlements over the two periods were also used to analyze the spatial differentiation of medical convenience levels within the study area.

**Results:**

The medical convenience levels of many settlements improved significantly. 80 settlements were greatly improved, while another 103 showed slight improvement.Areas with obvious improvement were distributed in clusters, and mainly located in the southwestern part of Yingjiang County, northern Longchuan County, eastern Lianghe County, and the region where Lianghe and Longchuan counties and Mang City intersect.

**Conclusions:**

Development of the road network was found to be a major contributor to improvements in MCI for mountain residents over the six-year period.

## Background

China has 55 different ethnic minority groups in addition to the Han Chinese. These groups are mainly located in eight of the country’s central and western provinces: Xinjiang, Qinghai, Tibet, Yunnan, Guizhou, Inner Mongolia, Guangxi, and Ningxia. Most ethnic minorities live in remote mountainous areas, deserts, or border areas [1, 2]. People from all 55 groups can be found in Southwest China, where their numbers amount to 35.55 million, accounting for 31% of China’s total ethnic minority population. This region hosts 30 ethnic minority groups with populations exceeding 5 000 each. Some of these groups are unique and indigenous to the region. Southwest China, centered on the Yunnan–Guizhou Plateau, has the highest global distribution density of ethnic minorities.

The southwestern region of China is situated at the junction of the Eurasian, Indian, and Pacific tectonic plates. The topography here is rugged and, it is one of the world’s steepest and most remote regions. The forbidding and complex terrains can present extreme inconvenience for everyday activities [3]. Access to basic medical and healthcare services has been recognized as “a basic right of the people” [4]. Medical convenience level is closely related to the lives and safety of ethnic minorities. It reflects the public service capacity in ethnic minority areas, and is an important assurance for maintaining unity and stability in those areas. Therefore, it is very important and necessary to evaluate medical convenience in ethnic minority areas of Southwest China.

The total area of Southwest China is about 2.35 millions square kilometers, where 19.8 million people live. According to China’s Health and Family Planning Statistical Yearbook (2015), there are totally 2153 first-class and above hospitals in mountain areas of Southwest China, which mainly distribute in urban regions. In this region, the hospitals are very rare at remote mountain areas and the distances from some settlements to a hospital are very long. Roads in mountain areas are highly vulnerable and complex, which are easily affected by topography and debris flow. It is difficult and inconvenient for residents in some remote mountainous regions to go to those hospitals.

Aiming to the medical convenience evaluation, this study focuses on road network analysis within ethnic minority areas, since the construction and development of road networks is one of the most important factors affecting medical convenience levels among ethnic minority communities in mountainous areas. Medical convenience index (MCI) and evaluation models are proposed from three perspectives: vulnerability, complexity, and accessibility. In 2012, in order to gain a comprehensive understanding of China’s current geographical conditions and meet the need of social and economic development and ecological civilization construction, the State Council of China released a notification to conduct the First Survey of National Geographical Conditions, also called the first China Geography Census (CGC), from 2013 to 2015. The CGC has two aspects of contents:(1)to survey the attributes of nature geographical factors, including the type, site, scope, area of topography, vegetation cover, water area, desert, open ground, and etc..(2)to survey the attributes of human geographical factors, including the classification, site and scope of transport network, settlements, geography units, and etc., which are closelyrelatedto human activities. The census provides the most authoritative, objective, and accurate geographical data for the present study.

## Related work

Current studies by Chinese and international scholars on medical and healthcare services for residents mostly focus on accessibility to hospitals, or uniform distribution of medical and healthcare facilities [5]. When analyzing medical accessibility, the minimum travel time/distance is commonly used because the requisite data are easily available, the calculation method is simple, and the results are readily understood [6–11].

In recent years, the most widely used method for studying medical accessibility and the balance between the demand and supply of medical and healthcare facilities is floating catchment area (FCA), and associated enhancements such as the two-step floating catchment area (2SFCA) method, enhanced two-step floating catchment area (E2SFCA) method and the three-step floating catchment area (2SFCA) method. FCA originated from spatial decomposition [12], and is a special case of gravity model [13]. The application and improvement of this method made calculations simpler and the results more rational [5, 6, 14–20]. Methods used in other studies included the gravity model [6, 16, 21] and kernel density estimation (KDE) [20, 22, 23]. Neutens (2015) [24] further analyzed the advantages and disadvantages of the aforementioned methods when studying medical accessibility. However, there remains a lack of research on road network vulnerability and its impact on residents’ medical convenience levels.

Despite numerous studies on road network vulnerability in the past two decades, the concept of vulnerability has yet to be clearly defined. It is often jointly explained with other related terms such as risk, reliability, flexibility, robustness, and resilience. Many scholars have also attempted to explore the inter-relationships between those terms [25–28]. A review of the literature indicated that research on road network vulnerability generally adopts one of the following perspectives:

i. The connectivity of the road network, taking into account its topological structure. For example, Kurauchi et al. (2009) [29] determined the critical index of each road segment by calculating the number of connecting links between journey origin and destination, thereby identifying critical segments in the road network. Rupi et al. (2015)[30]evaluated the vulnerability of mountain road networks by examining the connectivity between start and end points, before grading them.
ii. After a segment has deteriorated, the road network becomes disrupted or its traffic capacity declines. This reduces regional accessibility and leads to socioeconomic losses. These losses are used to determine and grade critical segments of the road network. For example, Jenelius et al. (2006) [27] ranked the importance of different roads based on their daily traffic volumes. Next, the impact of each road grading on traveling options and durations under various scenarios were simulated. Chen et al. (2007) [31] determined the vulnerability level of a road segment by the impact of its failure on regional accessibility, while Qiang and Nagurney (2008) [32]identified the relative importance and ranking of nodes and links within a road network by documenting the traffic volumes and behaviors of the network. Similarly, Jenelius and Mattsson (2012) [33] used traffic volumes to calculate the importance of the road network within each grid.
iii. The impact of a road network’s deterioration or obstruction for regional accessibility is assessed by simulating a particular scenario, for example the occurrence of a natural disaster or deliberate attack. The results provide decision support on the transportation and delivery of relief provisions, as well as disaster recovery efforts. Bono and Gutiérrez (2011) [34] analyzed the impacts of road network disruption caused by the Haiti earthquake on the accessibility of the urban area of ​​Port Au Prince.
iv. Computational models for evaluating road network vulnerability are subjected to optimization. Some scholars have focused on model optimization because they believe that the computational burden is very heavy when grading the vulnerability of each segment within the overall road network. On the basis of the Hansen integral index, Luathep (2011) [35] used the relative accessibility index (AI) to analyze the socioeconomic impacts subsequent to road network deterioration, which causes network disruption or reduction in traffic capacity. Next, the AIs of all critical road segments before and after network deterioration were compared for categorization and ranking. This method reduces both computational burden and memory requirements.

In the present study, road network vulnerability is determined by combining data on the paths of debris flow hazards with only links in the topological structure of a road network.

## Study area and data

### Study area

Yunnan Province is located at China’s southwestern border. It is far from the country’s political and cultural center, and is relatively undeveloped economically. The province’s terrain comprises mountains, plateaus, and highlands, with 94% of the total land area classified as mountainous. Yunnan has the most ethnic minority groups in China, account for 33.37% of the province’s total population. The National Autonomous Areas cover 276 700 km^2^ of land (70.2% of the province’s total land area). Prior to the founding of New China in 1949, many ethnic minorities living in Yunnan’s various border areas, ethnic minority areas, and alpine mountainous areas were still practicing slash-and-burn agriculture and suffered extreme poverty. There was also a serious lack of medical and healthcare services, with various diseases and illnesses being transmitted over successive years.

Dehong Prefecture is situated in the province’s western section and the southern foothills of the Gaoligong Mountains, and borders Myanmar to the southwest and northwest. The border has a length of 503.8 km and total land area is 11 500 km^2^. Mang City, the state capital, is 679 km from Kunming, the provincial capital. Currently, the prefectural jurisdiction includes two cities (Mang and Ruili), three counties (Longchuan, Yingjiang, and Lianghe), 50 townships, one sub-district office, 336 village committees/groups, and 40 urban neighborhood committees.

**Fig. 1:**
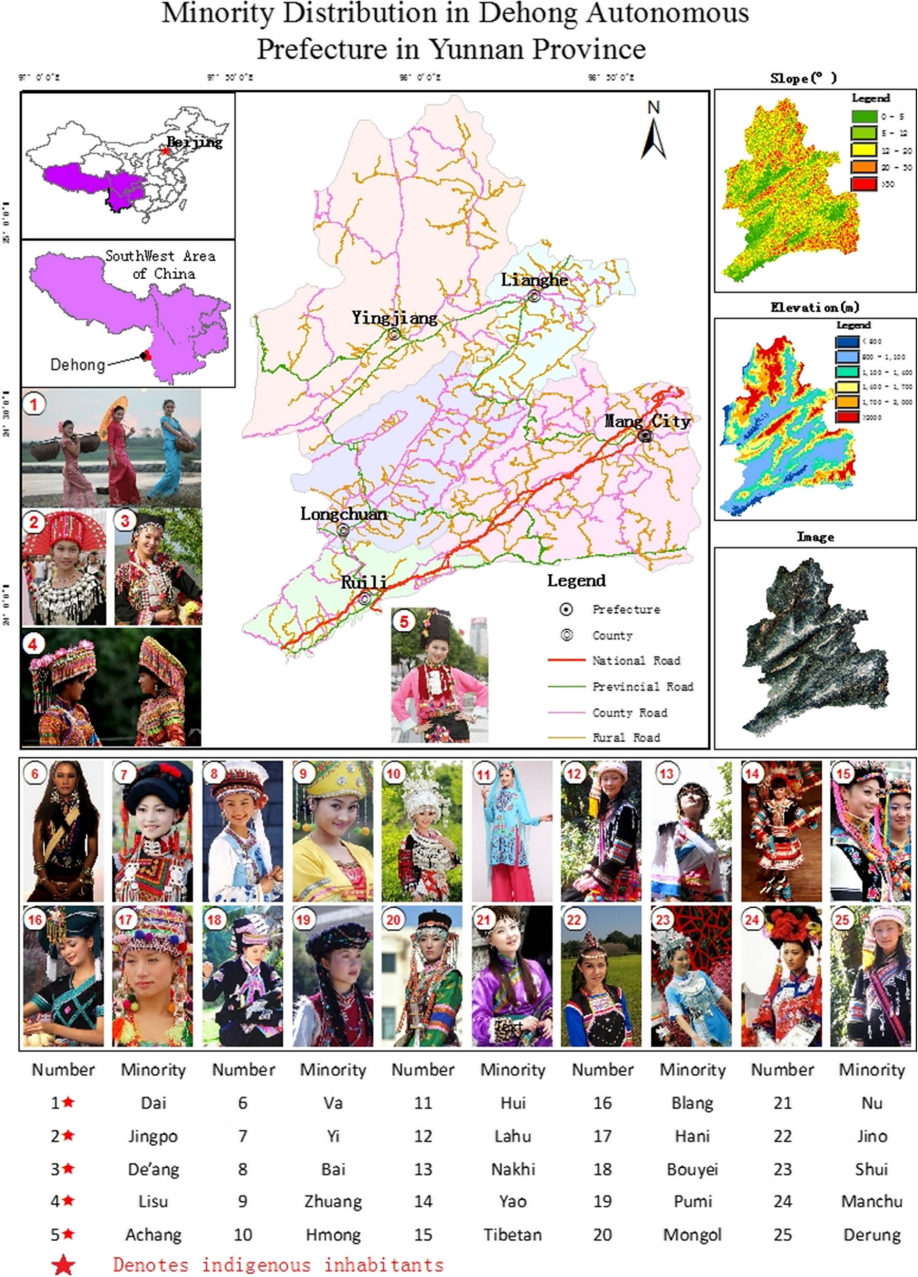
Study area and distribution of its ethnic minority groups

More than 30 ethnic minority groups live within the territory of Dehong Prefecture, including five indigenous groups (Dai, Jingpo, Lisu, Achang, and De’ang), and eight that are transboundary. The total population of the prefecture at the end of 2013 was 1.0678 million. Of this, 513 000 (48%) are from the Han ethnic group, while the remaining 52% consist of ethnic minority groups. The larger ethnic minority groups are the Dai, Jingpo, Achang, Lisu and De’ang, with population sizes of 337 300, 128 900, 28 400, 26 200, and 13 200, respectively. The other ethnic minority groups include the Yi, Bai, Zhuang, Hmong, Hui, Lahu, Nakhi, Yao, Tibetan, Blang, Bouyei, Hani, Va, Pumi, Mongol, Nu, Jino, Shui, Manchu, and Derung. Their distribution is shown in Fig. 1.

The entire territory of the prefecture is a low-latitude mountainous region that mainly comprises mountains of low and medium elevations. Mountainous areas form 89% of its total land area, while basins, dams, plains, and valleys make up the remaining 11%. The general elevation is 210.0–3 404.6 m, and most of the ridge line lies at an altitude of approximately 2 000 m. The mountains are mostly aligned northeast-to-southwest. The northeastern portion is high and steep; the southwestern portion is lower and gentler, with an inclined distribution towards the southwest. The valley and fault zone have the same orientation, with the former having developed on the latter.

**Fig. 2:**
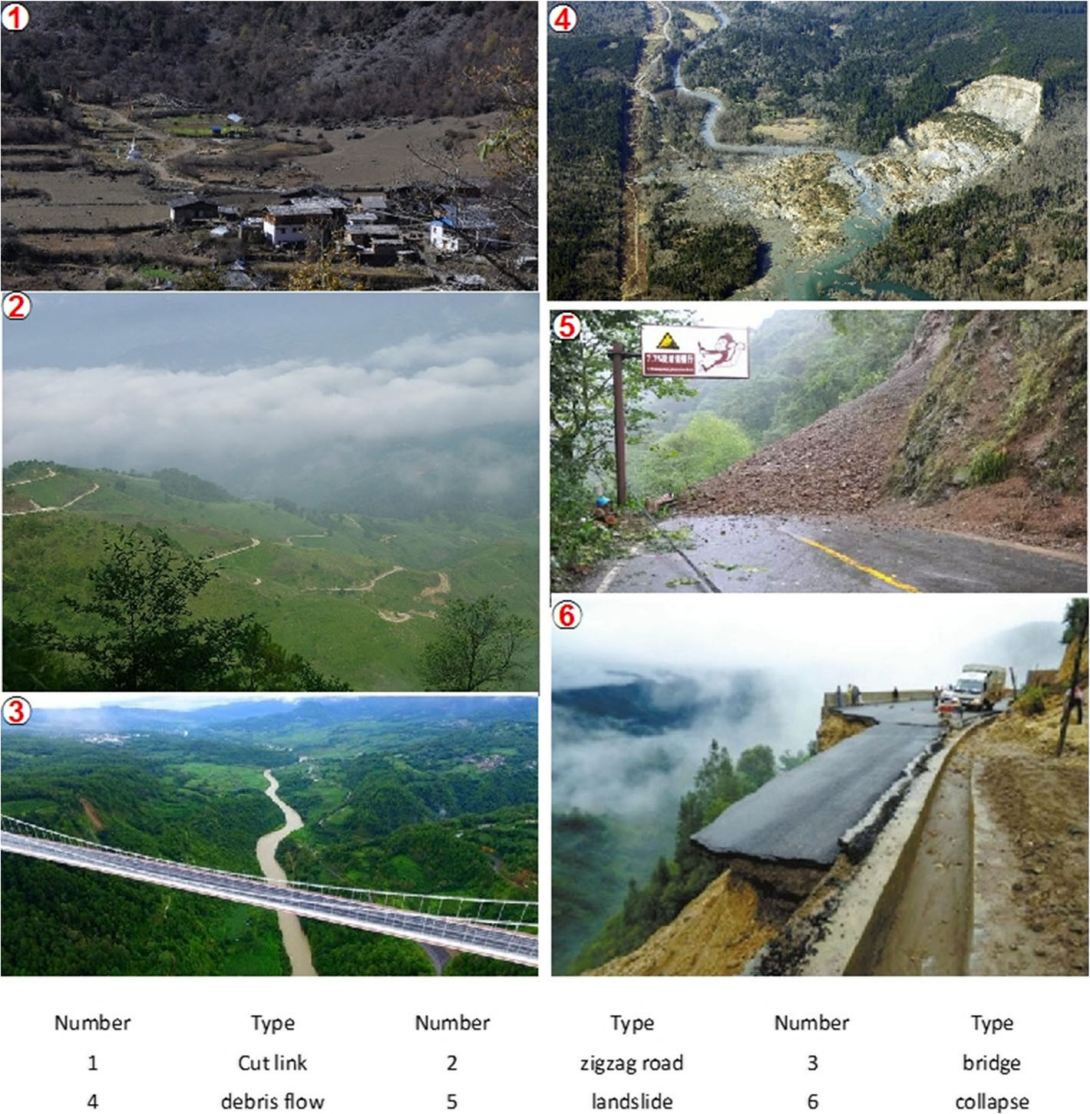
Examples of road network vulnerability within the study area

The road network within the prefecture has complex morphology. Some roads are built along gullies, while others straddle the two sides; some follow the mountains’ topography and meander upwards in a zigzag alignment, while others are cut into cliff faces. The roads have many bends and gradient changes, and there is a very high ratio of bridges and tunnels to roads. In addition, Dehong Prefecture is affected by the southwest monsoon from the Indian Ocean, and has a South Asian tropical monsoon climate with the year divided into dry and wet seasons. Precipitation is concentrated during May–October (accounting for 70–80% of annual rainfall), which often causes soil on the slopes to become saturated. Debris flows and landslides are easily triggered as a result of heavy rainfall or storms. Consequently, traffic capacities are reduced and roads may even be obstructed. Overall, road vulnerability is extremely high (Fig. 2).

### Data sources

#### Preprocessing of road network data

The study used road network data from two periods, obtained from 2009 Basic Surveying and Mapping data and 2015 CGC data, both of which used 1:50,000 road categorization systems. However, as a result of their different collection purposes, the latter has more abundant data on road networks. In order to accurately reflect the study area’s road situation in 2009, Google high resolution images were used. Google images coordinate (World Geodetic System 1984, WGS-84) were converted to China Geodetic Coordinate System 2000(CGCS 2000). These images were used to check and revise road networks at 2009.The use of the Google historic images helps to gather more minor roads at 2009.

#### Preprocessing of data on villages

The rural village committees within the study area were treated as ethnic minority settlements. The 2015 CGC provides the most current, complete, and accurate dataset for the study area, indicating 376 rural village committees. In the 2009 Basic Surveying and Mapping data at 1:50 000 scale, rural village committees were included under the general category of ground features without separate coding; furthermore, the data were also incomplete. Hence, this study extracted settlements from 2009 data for the Yunnan Provincial Administrative Division. These were supplemented by the 2015 CGC data for a complete set of data on the village committees in 2009, numbering 374 village/urban neighborhood committees.

#### Preprocessing of data on hospitals

Hospitals are graded according to their scales and service levels in China. When studying medical convenience levels, primary consideration was given to settlements’ accessibility to hospitals graded as first class and above as the delegations of all medical establishments. More small scale hospitals and clinics provide very low Medical services, most of which have no assistant doctor or high-level doctor [Hu et al., 2013]. Data on this category of hospital situated within the study area were obtained from the CGC data. In the 2009 Basic Surveying and Mapping data at 1:50 000 scale, hospitals were not included under the category of ground features. Hence, field research was conducted to ascertain whether qualifying hospitals shortlisted from the CGC already existed prior to 2009, have relocated, or have been reclassified.

#### Data on geological disaster spots

The locations of debris flow events within the research area were obtained from Yunnan Geological Survey Department (YGSD).The YGSD routinely monitors debris flow events since 2000, and surveyed the hidden debris flow disaster spots in Yunnan, China in 2011. The authors collected these data from Yunnan Bureau of Geological Survey.

#### Other data

Global Digital Elevation Model (GDEM) 30 M was downloaded from www.gscloud.cn. Villages located in the Yunnan Provincial Administrative District were obtained from annual handbooks published by the province’s Department of Civil Affairs. All data used in the research were listed in Table 1.

**Table 1:** Data Source

No.	Data	Year
1	1:50 000 DLG, DOM, DRG	2009
2	CGC	2015
3	Yunnan Provincial Administrative District	2009, 2016
4	Geological disaster spots	2011
5	GDEM 30 M	

*DLG* digital line graph, *DOM* digital orthophoto map, *DRG* digital raster graph

## Methods

### Evaluation model for medical convenience

This study examines the medical convenience levels of ethnic minority communities in mountainous areas. The study focuses on the medical and healthcare situation as this best represents the extent to which people’s livelihoods are protected. This was measured using the proposed MCI and computational model, which specifically considers ethnic minority residents of mountainous areas. The characteristics of the road network represent one of the most important factors affecting medical convenience levels. Hence, a model was constructed based on three aspects of the mountain road network: vulnerability, convenience, and complexity.

1 $$ \mathrm{A}={\sum}_{\mathrm{i}=1}^4{w}_i{x}_i $$

Here, *w*_i_ is the evaluation factor, and *x*_i_ is the factor’s weighting.

**Fig. 3:**
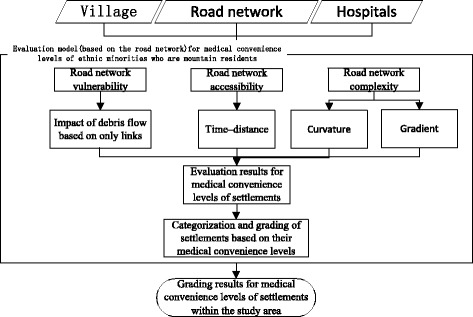
Evaluation process for medical convenience levels based on road networks

The model is innovative because it focuses on road network vulnerability in mountainous areas, and proposes a method of evaluating road network vulnerability by measuring the impact of debris flow based on cut highway links. The construction process for the model is shown in Fig. 3.

### Vulnerability

This study focused on ethnic minority areas in Southwest China. These are located mainly in mountainous areas, with road networks being constrained by the terrain. The resulting highway topology is a tree-like structure with many only links. Separately, there are also high incidences of debris flow hazards in these areas due to climate and geological structure.

**Fig. 4:**
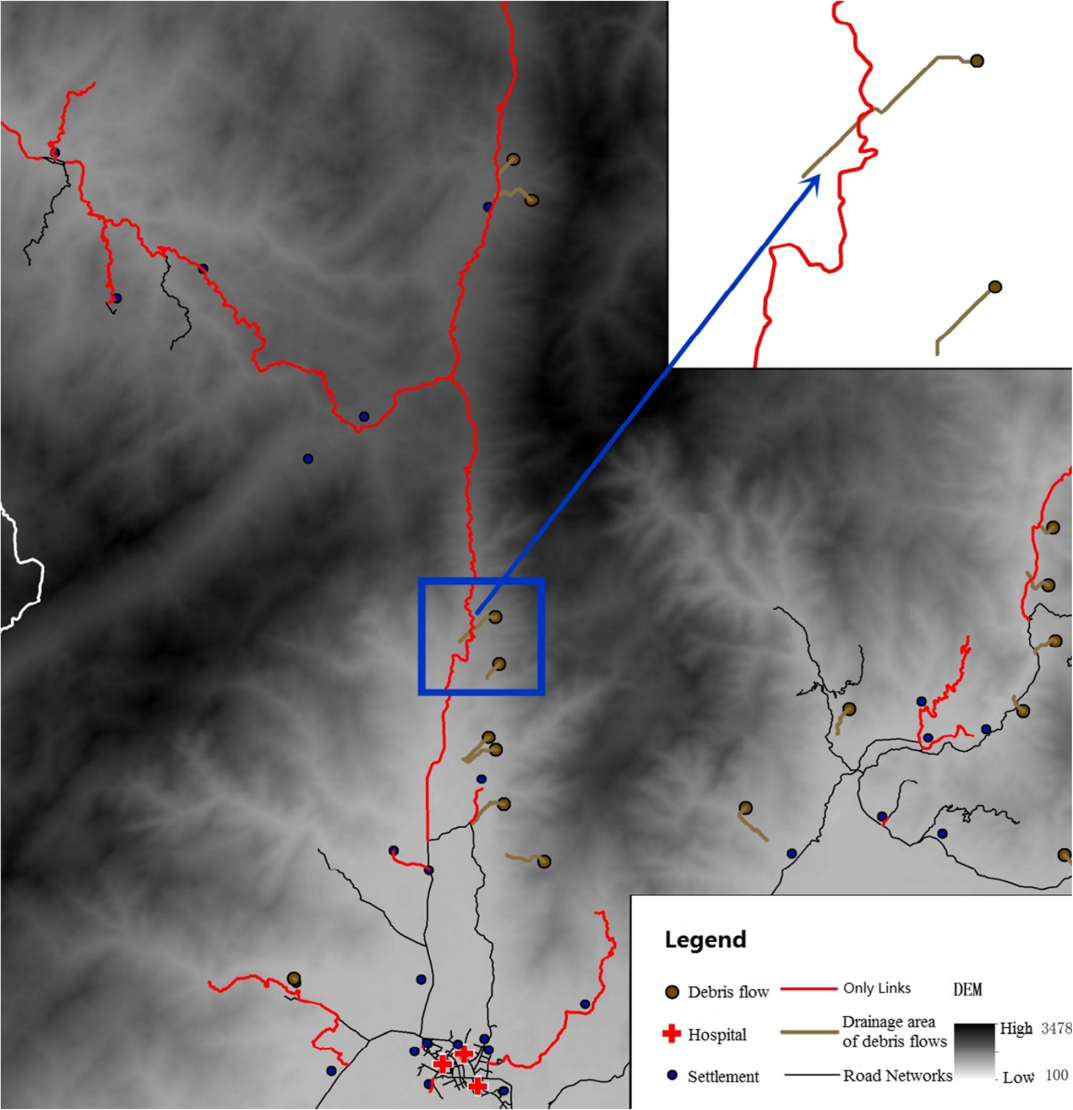
Flow chart for determining road network vulnerability

Following from the above, this study combined only links in the topological structure of the road networks with geographical locations prone to debris flows. This led to the proposed method of measuring the impact of debris flow based on cut highway links, which then facilitated the evaluation of road network vulnerability (Fig. 4).

#### Step 1: Obtain the shortest path

A settlement and its nearest qualifying hospital are used as journey start and end points respectively. Next, the road network is searched to determine the shortest path between the two points.

#### Step 2: Segmentation of the shortest path

The shortest path is divided into multiple segments based on the network nodes and road hierarchy.

#### Step 3: Check for only links

**Fig. 5:**
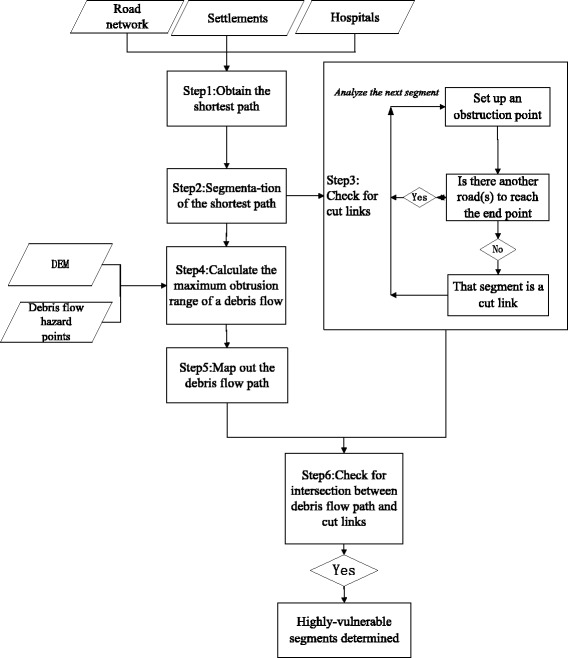
Schematic of only link-based debris flow

Obstruction points are set up for each segment in turn. When one particular segment is obstructed, the entire road network is searched to determine whether there is an alternative road(s) that allows travel between the start and end points. If there is no alternative route, then the obstructed segment is deemed to be an only link (Fig. 5). This process is repeated for the next segment, until all segments along the shortest path have been checked. Eventually, all only links along the shortest path are obtained.

#### Step 4: Calculate the maximum obtrusion range of a debris flow

Data on debris flow hazard locations are combined with GDEM data to simulate the direction of debris flows. Next, the equation for the maximum obtrusion range of debris flows, as proposed by [36], is used to constrain the flow distance. Finally, the drainage area of the debris flows and total extent of areas impacted are calculated.

2 $$ {L}_f=0.2024{\left(W\ast H\right)}^{0.1901} $$

In equation (2), *L*_*f*_ is the predicted maximum distance for a single obtrusion, *W* is the landslide volume of the debris flow sources, and *H* is the maximum relative height difference of the drainage area Table 2.

The landslide volume *W* of a debris flow source can be obtained from the corresponding flow level (see Table 2), which is based on attribute data for the debris flow hazard locations.

**Table 2:** Grading of debris flows

Flow level	Population affected	Corresponding scale	Volume of flow (10^3^ m^3^)
A	>1000	Huge	≥ 50
B	100–1000	Large	20–50
C	10–100	Medium	2–20
D	0–10	Small	< 2

#### Step 5: Map debris flow paths

Simulation of debris flow direction involves superimposing the location of the debris flow hazard onto the GDEM using the same coordinate system. The GDEM pixel for the location of the debris flow hazard is then designated as the center, before the elevation values of the eight pixels surrounding the central pixel are obtained. Of the eight elevation values, only those lower than the central pixel are selected. From the selection, the pixel with the lowest elevation becomes the subsequent point in the direction of debris flow. Mapping is terminated when the length of the flow path exceeds the value of *L*_*f*_ (maximum obtrusion distance) as calculated in Step 4. This process is repeated for all debris flow hazard locations, until the paths of the overall debris flows have been mapped out.

#### Step 6: Check for intersection between debris flow path and only links

After the entire flow path is mapped, the model checks whether it intersects any only links. If the flow path intersects the road link, the debris flow poses a potential threat to those only links, making the affected segments extremely vulnerable. This in turn greatly affects the medical convenience level of settlements located along those highway segments.

### Accessibility

The assessment of medical convenience levels should consider the accessibility of settlements to hospitals. Accessibility can be measured using many methods, including spatial distance, time–distance, economic distance, and cumulative opportunities. Regardless of the model used, transportation costs between the point of demand and a facility must be calculated.

Since the topography of the study area comprises mostly mountains and plateaus, the hierarchy and design speed of roads have a major impact on travel times between an origin and destination. Medical convenience can make the difference between life and death, and time is critical during emergencies. Thus, the time–distance method was adopted in this study as the proxy for accessibility of settlements to hospitals graded first class and above. Using this method, shorter travel time represents greater convenience for residents to make use of medical and healthcare facilities. This reflects that such facilities having been planned and established via a more rational approach, as well as a higher level of medical convenience.

### Complexity

#### Curvature

When designing a non-linear road, the degree of curvature reflects the number and type of bends in the road. The larger the curvature, the more complex is the road geometry, and the lower its convenience level. This study employed curvature to describe the amount of bends in the road network. The entire road is treated as one curve, and the curvature is calculated individually for each of its segment. The cumulative curvatures of all segments form the overall curvature of the road, as follows:

3 $$ \mathrm{k}={\sum}_1^{i=n}\frac{\Delta {\varphi}_i}{\Delta {s}_i} $$

where *∆φ*_*i*_ is the change in angle between three adjacent points on the curve; 0° < <*∆φ*_*i*_< < 180°; and *∆s*_*i*_ is the arc length of the three adjacent points.

#### Gradient

The gradient of a road has the same impact on its capacity as curvature, with excessive gradients greatly reducing speeds and thereby increasing the travel time to medical facilities. The threshold gradient for general roads is 11°. Steeper gradients impose significant reductions in a road’s capacity, making travel much more difficult. Gradient is the ratio of the elevation difference between two points to the horizontal distance between those points. The overall gradient of a road is the sum of those gradients for all its segments, expressed as follows:

4 $$ \mathrm{i}={\sum}_{\mathrm{k}=1}^{\mathrm{n}}\raisebox{1ex}{${h}_k$}\!\left/ \!\raisebox{-1ex}{${l}_k$}\right.\times 100\%\kern0.5em $$

where *h* is elevation difference and *l* is horizontal distance.

The setting of weightings for both gradient and curvature is similar: only the impact on convenience resulting from vulnerability (as caused by the gradient) is considered.

### Setting weightings the various indices

For the setting of weightings, prior knowledge of the study area was combined with scoring by experts. The weighting of each index in the model was determined after repeated experiments (Table 3).

**Table 3:** Weighting of each index in the model

	Index	Threshold	Weighting
Vulnerability	Only link-based debris flow	Only link	0.2
		Debris flow	0.2
		Combined effect of only link and debris flow	Infinite
Accessibility	Time–Distance (min)	0–4	0.05
		4–24	0.12
		24–54	0.25
		> 54	0.4
Complexity	Curvature	0–0.1	0.01
		0.1–0.2	0.06
		> 0.2	0.1
	Gradient	0–0.03	0.005
		0.03–0.05	0.02
		0.05–0.08	0.05
		0.08–0.11	0.08
		> 0.11	0.1

### Evaluation and grading of medical convenience

The model for evaluating medical convenience in mountainous areas was used to compute the MCIs of the various settlements. The results were then compared with the actual medical and healthcare situations on the ground. The MCI was divided into five levels as shown in Table 4.

**Table 4:** Classification standards for medical convenience levels in the study area

Medical convenience level	A	B	C	D	E
	Good	Quite Good	Average	Quite Poor	Poor
MCI	< 0.18	0.18–0.34	0.34–0.54	0.54–0.78	0.78–1

## Results and discussion

Data for Yunnan Province from the first CGC in 2015 and basic surveying data from 2009 were used to construct a model of medical convenience levels in mountainous areas. The levels for 2015 and 2009 were then evaluated separately.

### Spatial differentiation in medical convenience levels

#### Spatial differentiation in 2009

**Fig. 6:**
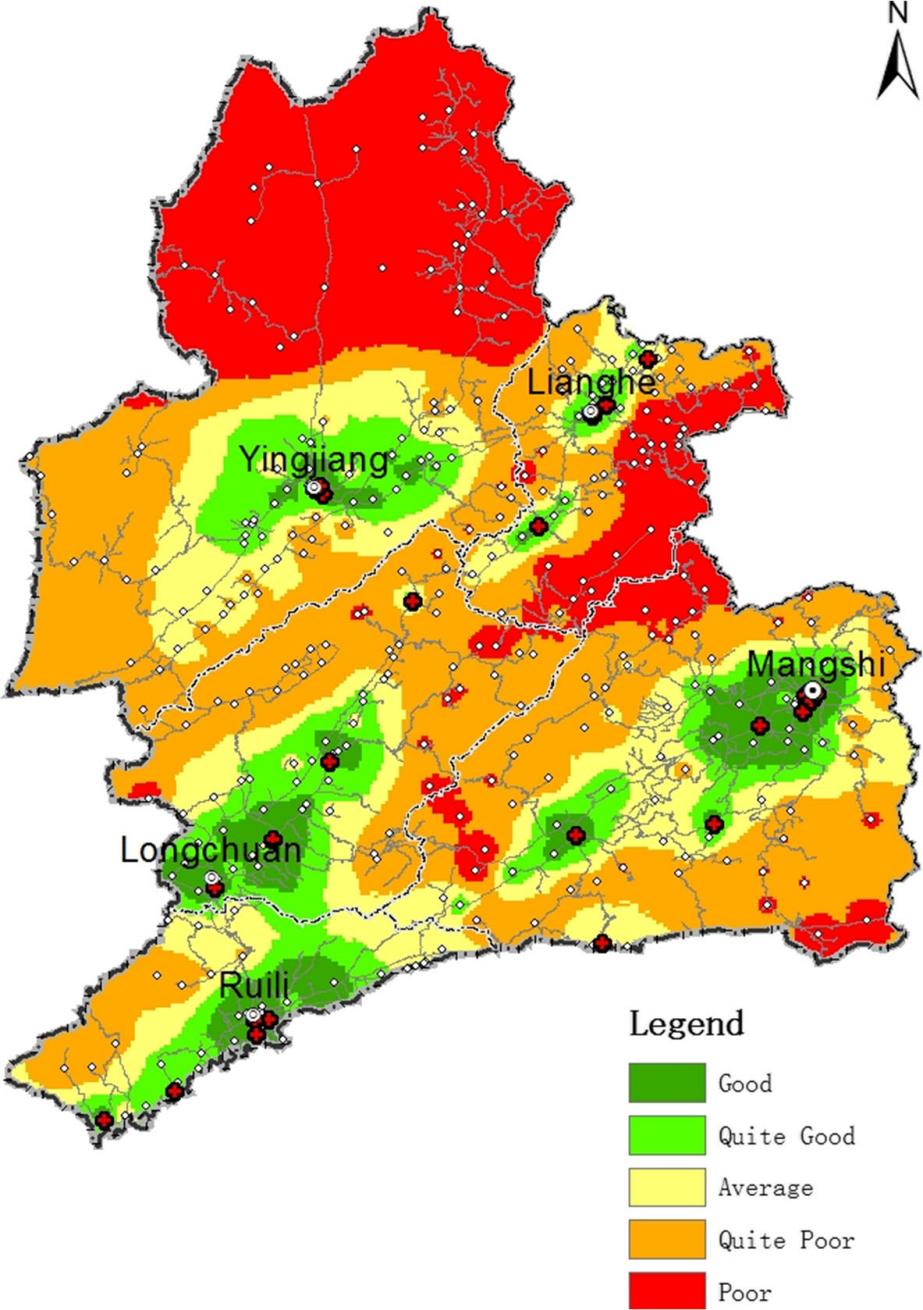
Medical convenience levels of Dehong Prefecture, 2009

The study area contained 374 settlements in 2009, of which 87 were graded as level A. These were mostly concentrated in the urban areas of Yingjiang, Longchuan, Ruili, and Lianghe counties and in Mang City. Specifically, these are locations where the respective county or city governments are sited, and are areas with well-developed road networks. Their spatial distributions were clustered, as shown by the areas colored dark-green in Fig. 6. There were 44 and 49 settlements graded levels B and C, respectively (light-green and yellow areas respectively in Fig. 6). These were mostly located along the road networks and radiated outwards from the urban areas and their surroundings.

There were 88 settlements classified as level D and 106 level E. These areas (colored orange and red respectively in Fig. 6) were mainly located in remote mountainous areas far from towns and cities, and had poorer road networks. Examples include the northern part of Yingjiang County, southern Mang City, and the region where Lianghe and Longchuan counties and Mang City intersect. Settlements graded quite good/good and quite poor/poor accounted for 34.5% and 52.5% respectively of the total number of settlements within the study area (see Table 5).

**Table 5:** Ranked medical convenience levels of settlements in the study area, 2009–2015

Year	Level	Number of settlements	Percentage
2009	A	87	22.5%
	B	44	12%
	C	49	13%
	D	88	24%
	E	106	28.5%
	Total	374	
2015	A	156	41.5%
	B	52	13.8%
	C	62	16.5%
	D	63	16.8%
	E	43	11.4%
	Total	376	

Ruili City and the surrounding county had generally good levels of medical convenience: Of its 40 settlements, 24 (60%) were graded quite good/good, far exceeding the average level for the study area. Ever since Ruili withdrew from the county and was established as a city in 1992, rapid developments in border trade led to continuous improvements in the quality of its basic infrastructure (including municipal administration, transportation, power supply, communication, water conservation, education, and sanitation). Its road network density, number of road nodes, and distribution of hospitals were superior to those of the other counties (cities).

Ranked next were Mang City and Longchuan County, for which 38% and 39% of settlements were graded quite good/good for medical convenience. These levels were similar to that for Dehong Prefecture as a whole. Mang City is the capital of Dehong Prefecture, and the roads surrounding the capital are in good condition. Longchuan County is adjacent to Ruili City. Road conditions in its southwestern part (near Ruili City) are good, such that the medical convenience level matches that of the overall prefecture.

Lianghe and Yingjiang counties are located far from the open frontier ports and the prefectural capitals, where economic conditions are better. The northern part of Yingjiang County and southeastern Lianghe County are also further from their respective county towns. The road networks and provision of hospitals in those two areas are relatively less developed. These factors are detrimental to medical convenience levels, with only 24% and 26% of their respective county settlements graded quite good/good, and 66% and 60% respectively graded quite poor/poor (Fig. 6).

#### Spatial differentiation in medical convenience levels of Dehong prefecture, 2015

**Fig. 7:**
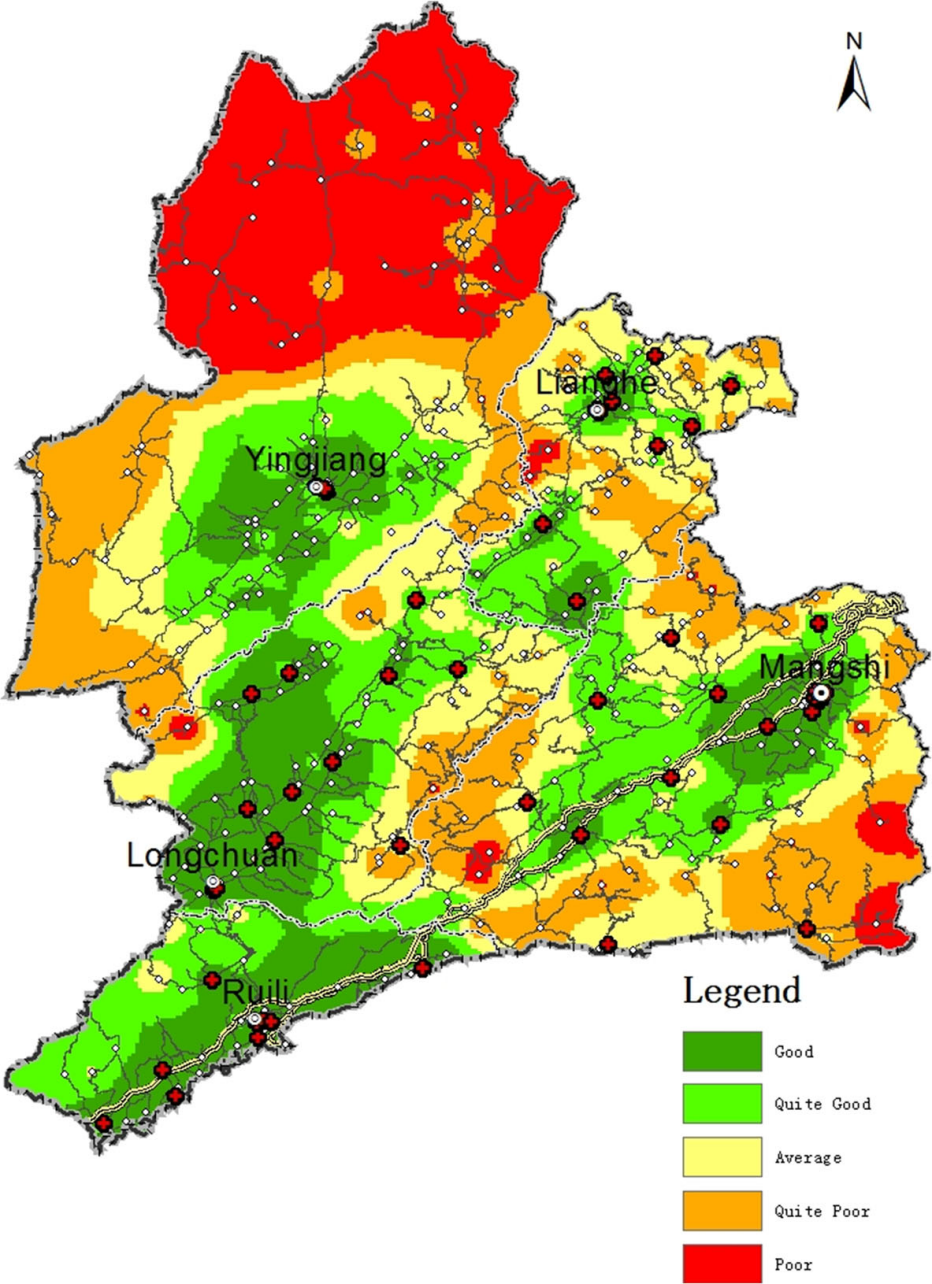
Medical convenience levels of Dehong Prefecture, 2015

The number of settlements in the study area increased to 376 in 2015. Of these, 156 and 52 were graded Levels A and B (colored dark-green and light-green respectively in Fig. 7). These jointly accounted for 55.3% of settlements within the study area. Settlements with better MCIs have stripe-like distributions, and are located on both sides of highways/higher-hierarchy roads and in areas with denser road networks. There were 62, 52, and 43 settlements graded C, D, and E, respectively. Those graded quite poor/poor jointly accounted for 28.2% of settlements within the study area, and were mainly distributed in areas with lower-hierarchy roads and simple road network structures (areas colored red and orange respectively in Fig. 7).

At the county/city level, in 2015, Ruili City maintained its top position for medical convenience: 85% of its settlements were categorized as levels A or B combined, and the remaining 15% were graded level C. Settlements that were previously graded levels D and E had been eradicated. Such progress cannot be dissociated from the city being located at the open frontier, and its maintenance of consistently high economic growth rates.

Longchuan County ranked second: 68% of its settlements were graded quite good/good, which was far better than the average for the overall study area. These settlements mostly formed strip-like distribution patterns. Construction of new roads in recent years has greatly improved the county’s overall road network, resulting in better levels of medical convenience than those of the remaining three counties/cities.

### Changes in medical convenience levels

Quantitative evaluation was conducted of changes in medical convenience levels within the study area between 2009 and 2015. The results were categorized as follows: unchanged, slightly improved (by one grade), and greatly improved (by two grades or more).

#### Changes in medical convenience levels of Dehong prefecture at the prefectural level

**Fig. 8:**
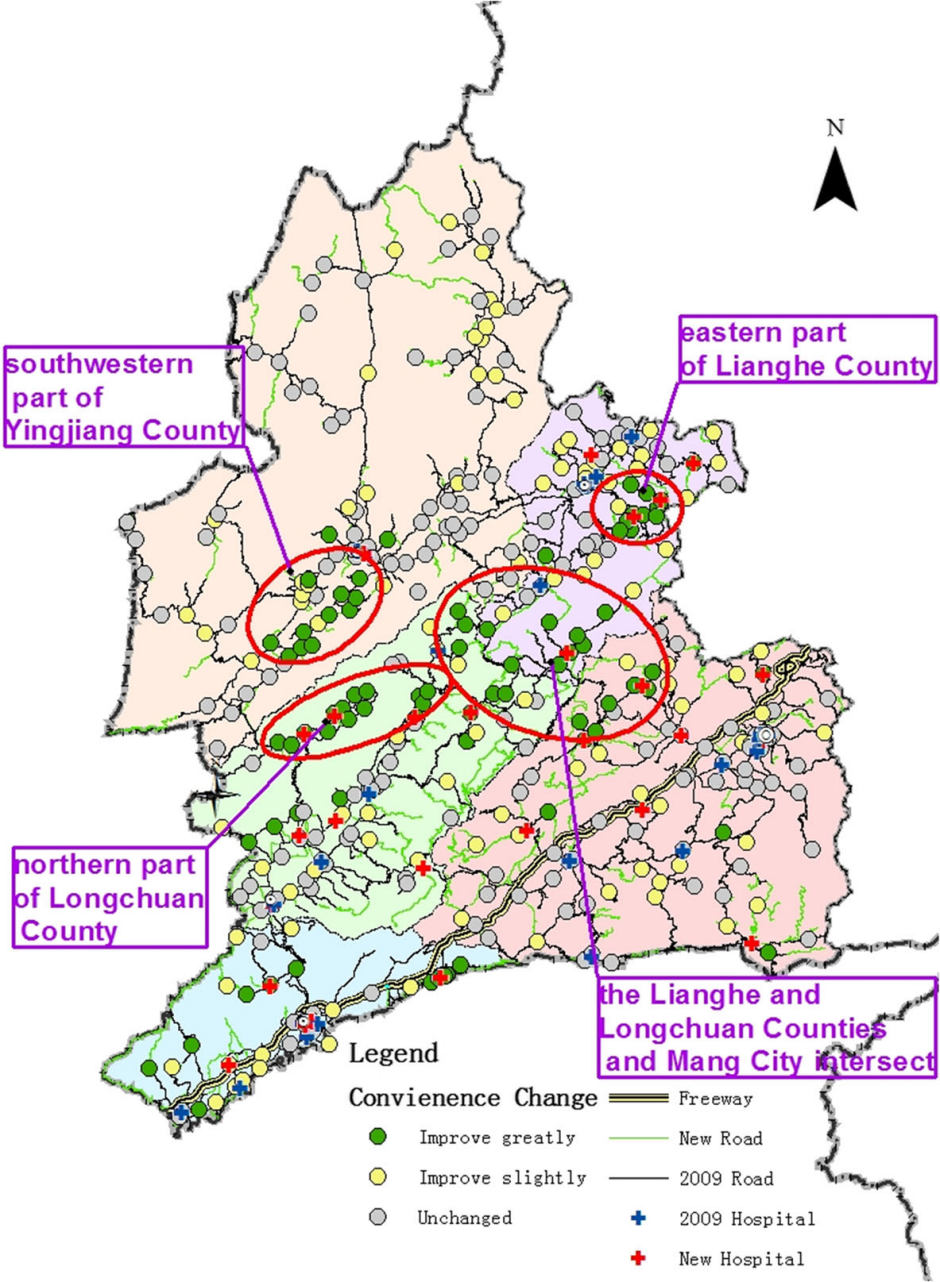
Changes in medical convenience levels of Dehong Prefecture at the prefectural level, 2009–2015

The number of settlements in the study area increased from the original 374 to 376. Evaluation of medical convenience levels showed that the situation was greatly improved for 80 settlements, while another 103 showed slight improvement. Areas with obvious improvement were distributed in clusters, and mainly located in the southwestern part of Yingjiang County, northern Longchuan County, eastern Lianghe County, and the region where Lianghe and Longchuan counties and Mang City intersect (Fig. 8). Improvements in medical convenience levels resulted from the development and construction of basic infrastructure: 1 401 new roads were constructed in 2009–2015, which added 1 044 km to the network length. Overall, road coverage had increased substantially.

#### Changes in medical convenience levels of Dehong prefecture at the county level

**Fig. 9:**
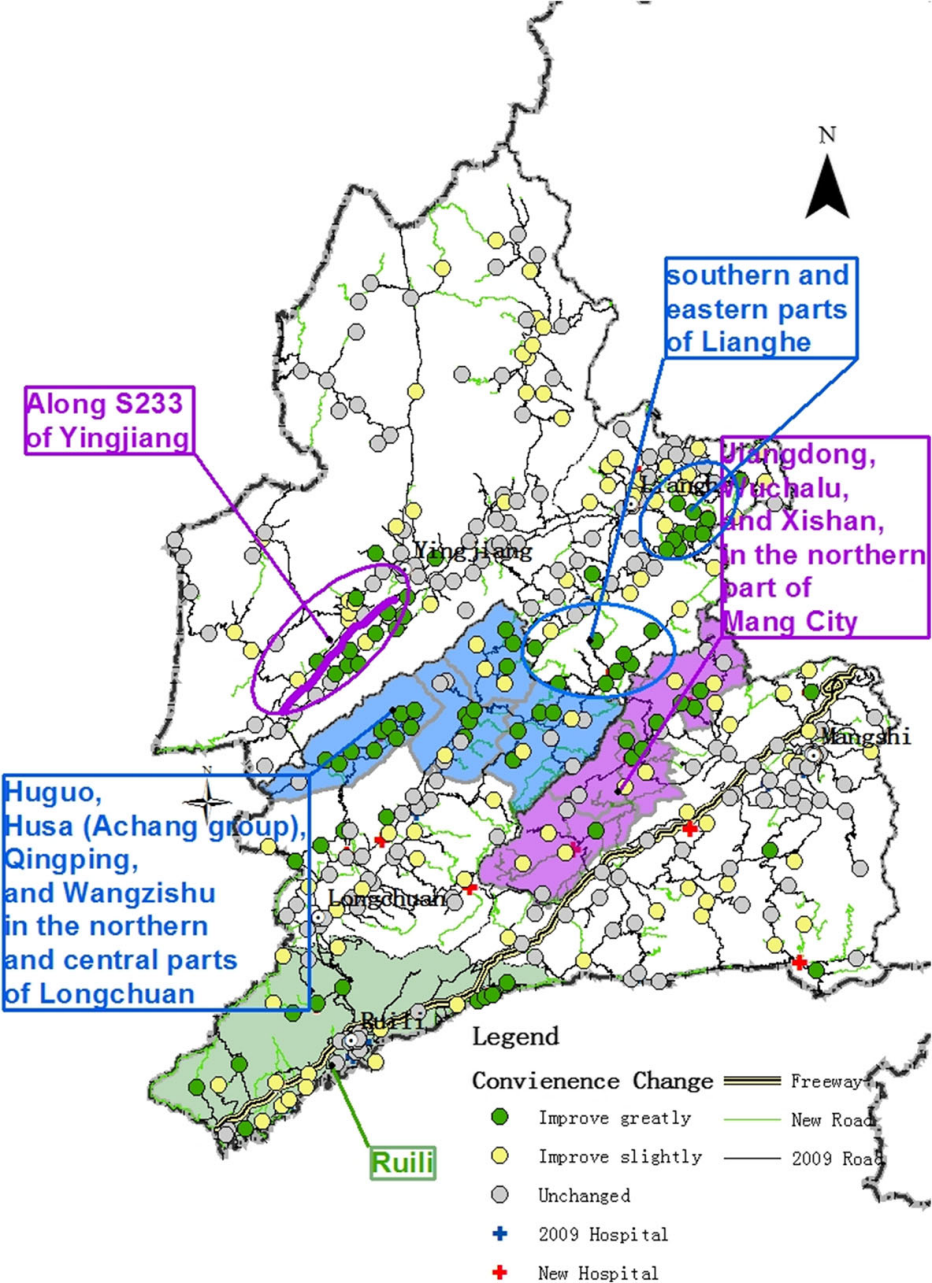
Changes in medical convenience levels of Dehong Prefecture at the county level, 2009–2015

Although there was an overall improvement in medical convenience levels in Dehong Prefecture over the six years, the situation for individual counties and cities within the prefecture varied. Longchuan County showed the most obvious improvement, with 25 settlements having improved greatly and 15 slightly. These were concentrated in the northern and central parts of the county, and include the following townships: Huguo, Husa (Achang group), Qingping, and Wangzishu (areas colored blue in Fig. 9).

For Lianghe County, 20 settlements showed greatly improved and 22 slightly improved medical convenience levels. These were mainly concentrated in the southern and eastern parts of the county (areas enclosed by blue ovals in Fig. 9). In comparison, in Yingjiang County, 13 settlements showed great improvement and 26 slight improvement. Most of these were distributed along S233 (areas enclosed by purple ovals in Fig. 9).

In Mang City, 11 settlements improved greatly and 27 improved slightly. These areas were in the townships of Jiangdong, Wuchalu, and Xishan, all located in the northern part of the city (areas colored purple in Fig. 9). Ruili City already had generally good medical convenience levels. On that foundation, 11 and 13 of its settlements had great and slight improvements, respectively (areas colored green in Fig. 9).

#### Areas with no changes in medical convenience levels and associated causes

Some areas did not see any significant improvements in medical convenience levels over the six years, such as the northern and western parts of Yingjiang County, and southern Mang City. The topography of these areas is complex, and their road networks are poorly developed and highly vulnerable. These pose objective difficulties when attempting to improve accessibility for people living in these areas.

## Conclusion

The following conclusions are drawn from the analysis:

i. The validity of the proposed model was demonstrated by evaluating the medical convenience levels of more than 370 settlements within the study area. The model was separately applied to data from 2009 and 2015, indicating that ethnic minority communities located in areas with comparatively rapid economic development, gentler terrain, and better-developed road networks enjoyed higher levels of medical convenience. Conversely, convenience levels were low in areas with steep terrain, frequent natural hazards, and less-developed road networks. The proposed model and index are also applicable to other parameters concerning the protection of ethnic minority livelihoods in mountainous locations, such as convenience levels for educational services and public security.
ii. There was a six-year interval between the two data sets. Comparison of each settlement between the two periods showed that medical convenience levels had generally improved for ethnic communities located in the mountainous parts of the study area. Areas with obvious improvements mainly benefited from newly-constructed road networks. It can therefore be concluded that the development of road networks has a substantial impact on medical convenience levels among ethnic communities in these mountainous areas.
iii. The findings reflect the positive effects of road networks for protection of people’s livelihoods. Thus, when attempting to improve quality of life and livelihoods for ethnic minority communities, in addition to constructing new roads to reduce the vulnerability of road networks, efforts should also be made to improve the condition of existing roads, as well as reducing the complexity and improving the accessibility of roads.

The core of this study is to model medical convenience based on roadnetwork vulnerability in mountainous areas. In this study, we take Dehong Autonomous Prefecture, Yunnan Province, China, as a case study area. This model may be applied in other mountainous areas with the characteristic of road network vulnerability. On the other hand, this model may also be applied not only for the calculation of the medical convenience from settlements to hospitals, but also for the educational convenience from settlements to school. We also think, if at non-mountainous area(with developed road network or no debris flow), the medical convenience evaluation work do not need this model.

When constructing a road network model for evaluating medical convenience levels in ethnic minority areas, the main foci of this paper were static factors such as network vulnerability, accessibility, and complexity. The findings can inform the construction and improvement of future road networks. In subsequent studies, other factors affecting medical convenience levels will also be considered, including levels of road congestion. Data on road conditions, which reflect road congestion, will be incorporated for a more objective and comprehensive evaluation of medical convenience levels in ethnic minority areas, with the aim of providing targeted recommendations for the development and optimization of road networks in these areas.

## Abbreviations

AI: Accessibility Index
CGC: China Geography Census
FCA: Floating Catchment Area
GDEM: Global Digital Elevation Model
KDE: Kernel Density Estimation
MCI: Medical Convenience Index
YGSD: Yunnan Geological Survey Department

## References

[1] Qin X (1998). Distribution characteristics and changes of economic social development of minority nationality in border area of our country. Natural Sciences Journal of Harbin Normal University.

[2] Guan Y. China's ethnic minority groups distribution and its characteristics. *Nationalities* Forum. 1996;3:19–23.

[3] Ji X, An S (2010). A study of the economic pattern shift in western China since the its large-scale development. Journal of Southwest University (Social Sciences Edition).

[4] Cheng J, Cheng J, Lu Y, Huang ZH, Cao F (2015). Spatial inequity in access to healthcare facilities at a county level in a developing country: a case study of Deqing County, Zhejiang, China. Int J Equity Health.

[5] Hu R, Dong S, Zhao Y, Hu H, Li Z. Assessing potential spatial accessibility of health services in rural China: a case study of Donghai County. Int J Equity Health. 2013;12(1):414–4. 10.1186/1475-9276-12-35 PMC3747861 23688278

[6] Apparicio P, Abdelmajid M, Riva M, Shearmur R (2008). Comparing alternative approaches to measuring the geographical accessibility of urban health services: distance types and aggregation-error issues. Int J Health Geogr.

[7] Delamater PL, Messina JP, Shortridge AM, Grady SC (2012). Measuring geographic access to health care: raster and network-based methods. Int J Health Geogr.

[8] Khan OA, Skinner R. Mapping accessibility to general practitioners. Geographic Information Systems & Health Applications. 2003:209–308.

[9] Dewulf B, Neutens T, De Weerdt Y, Van de Weghe N (2013). Accessibility to primary health care in Belgium: an evaluation of policies awarding financial assistance in shortage areas. BMC Fam Pract.

[10] Schuurman N, Fiedler RS, Grzybowski SCW, Grund D (2006). Defining rational hospital catchments for non-urban areas based on travel-time. Int J Health Geogr.

[11] Tanser F, Gijsbertsen B, Herbst K (2006). Modelling and understanding primary health care accessibility and utilization in rural South Africa: an exploration using a geographical information system. Soc Sci Med.

[12] Radke J, Mu L. Spatial decompositions, modeling and mapping service regions to predict access to social programs. *Geographic* Information Science. 2000;6(2):105–12.

[13] Joseph A, Bantock P (1982). Measuring potential physical accessibility to general practitioners in rural areas: a method and case study. Soc Sci Med.

[14] Bell S, Wilson K, Shah TI, Gersher S, Elliott T (2012). Investigating impacts of positional error on potential health care accessibility. Spat Spatiotemporal Epidemiol.

[15] Langford M, Higgs G (2006). Measuring potential access to primary healthcare services: the influence of alternative spatial representations of population. Prof Geogr.

[16] Luo W, Wang F (2003). Measures of spatial accessibility to health care in a GIS environment: synthesis and a case study in the Chicago region. Environ Plann B.

[17] Luo J (2014). Integrating the huff model and floating catchment area methods to analyze spatial access to healthcare services. Trans GIS.

[18] Mao L, Nekorchuk D (2013). Measuring spatial accessibility to healthcare for populations with multiple transportation modes. Health Place.

[19] Wan N, Zou B, Sternberg TA (2012). Three -step floating catchment area method for analyzing spatial access to health services. Int J Geogr Inf Sci.

[20] Yang DH, Goerge R, Mullner R (2006). Comparing GIS-based methods of measuring spatial accessibility to health services. J Med Syst.

[21] Haynes R, Lovett A, Sunnenberg G (2003). Potential accessibility, travel time, and consumer choice: geographical variations in general medical practice registrations in eastern England. Environ Plann.

[22] McLafferty S, Grady S (2004). Prenatal care need and access: a GIS analysis. J Med Syst.

[23] Spencer J, Angeles G (2007). Kernel density estimation as a technique for assessing availability of health services in Nicaragua. Health Serv Outcome Res Methodol.

[24] Neutens T (2015). Accessibility, equity and health care: review and research directions for transport geographers. J Transp Geogr.

[25] Berdica K (2002). An introduction to road vulnerability: what has been done, is done and should be done. Transp Policy.

[26] D'Este GM, Taylor MAP: Network vulnerability: An approach to reliability analysis at the level of national strategic transport networks. Network Reliability of Transport. *Proceedings of the 1st INSTR* 2003*.*

[27] Jenelius E, Petersen T, Mattsson LG (2006). Importance and exposure in road network vulnerability analysis. Transport Res A-Pol.

[28] Mattsson LG, Jenelius E (2015). Vulnerability and resilience of transport systems – a discussion of recent research. Transport Res A-Pol.

[29] Kurauchi F, Uno N, Sumalee A, Seto,Y: Network evaluation based on connectivity vulnerability. In: Lam WHK, Wong SC, Hong KL editors. Transportation and traffic theory 2009: golden Jubilee. HongKong: Academic; 2009.p.637–649.

[30] Rupi F, Bernardi S, Rossi G, Danesi A (2015). The evaluation of road network vulnerability in mountainous areas: a case study. Netw Spat Econ.

[31] Chen A, Yang C, Kongsomsaksakul S, Lee M (2007). Network-based accessibility measures for vulnerability analysis of degradable transportation networks. Netw Spat Econ.

[32] Qiang Q, Nagurney A (2008). A unified network performance measure with importance identification and the ranking of network components. Optim Lett.

[33] Jenelius E, Mattsson LG (2012). Road network vulnerability analysis of area-covering disruptions: a grid-based approach with case study. Transport Res A-Pol.

[34] Bono F, Gutiérrez E (2011). A network-based analysis of the impact of structural damage on urban accessibility following a disaster: the case of the seismically damaged port au prince and Carrefour urban road networks. J Transp Geogr.

[35] Luathep P, Sumalee A, Ho HW, Kurauchi F (2011). Large-scale road network vulnerability analysis: a sensitivity analysis based approach. Transportation.

[36] Zhu J, Chang M, Ding J, Qi X (2012). Prediction for hazard zones of rainstorm induced debris flows in the Wenchuan earthquake epicenters. J Eng Geol.

